# Feasibility of Preserving No. 5 and No. 6 Lymph Nodes in Gastrectomy of Proximal Gastric Adenocarcinoma: A Retrospective Analysis of 395 Patients

**DOI:** 10.3389/fonc.2022.810509

**Published:** 2022-02-28

**Authors:** Xiao Yang, Yanan Zheng, Runhua Feng, Zhenggang Zhu, Min Yan, Chen Li

**Affiliations:** ^1^ Department of General Surgery, Ruijin Hospital, Shanghai Jiao Tong University School of Medicine, Shanghai Institute of Digestive Surgery, Shanghai Key Laboratory of Gastric Neoplasms, Shanghai, China; ^2^ Department of Gastrointestinal and Hernia Surgery, First Affiliated Hospital of Kunming Medical University, Kunming, China

**Keywords:** lymph node metastasis, predictive factors, gastrectomy, lymph nodes no. 5 and no. 6, proximal gastric cancer

## Abstract

**Objective:**

The extent of regional lymphadenectomy for proximal gastric cancer (PGC) has remained a controversy and a matter of considerable debate for a long time. We retrospectively analyzed the clinicopathological features to investigate the predictive factors for No. 5 and/or No. 6 lymph node metastases (LNMs) and evaluate the feasibility of performing proximal gastrectomy (PG) with preservation of No. 5 and/or No. 6 lymph nodes for these patients.

**Method:**

Patients who had undergone total gastrectomy plus D2 lymphadenectomy in the Department of Gastrointestinal Surgery, Ruijin Hospital, Shanghai Jiao Tong University, School of Medicine, from January 2008 to December 2017 were retrospectively collected and analyzed.

**Results:**

Among the 395 eligible patients in our study, 34 patients (8.61%) had No. 5 and No. 6 LNM. The degree of differentiation, Borrmann classification, vascular or perineural invasion, tumor diameter, depth of invasion, and other perigastric LNM were associated with No. 5 and/or No. 6 LNM. Multivariate analyses showed that tumor diameter ≥4 cm, No. 4 LNM positive, and No. 7, No. 8, No. 9 LNM positive were independent risk factors of No. 5 and/or No. 6 LNM. No. 5 and/or No. 6 LNM was not observed in the 105 patients who were staged from T1 to T3 and were found to be without independent risk factors.

**Conclusion:**

The metastatic rate of No. 5 and/or No. 6 lymph node of the proximal gastric adenocarcinoma was closely associated with the diameter of the tumor and other perigastric LNMs. It is feasible to preserve No. 5 and No. 6 lymph nodes with PG for the T1–T3 patients with lower risk of No. 5 and/or No. 6 LNM.

## Introduction

According to the GLOBOCAN 2020, gastric cancer ranked fifth for incidence and the fourth leading cause of cancer death worldwide, with the highest incidence rate in Eastern Asia (1). As the incidence of gastric cancer in the antrum decreases, the frequency of proximal gastric cancer (PGC) in the upper third has been increasing in the past few decades (2). Gastrectomy has undergone tremendous changes in surgical procedures and surgical indications. Distal gastrectomy and total gastrectomy (TG) with locoregional radical resection by adequate lymphadenectomy, involving resection of the pylorus, lead to long-term problems of dumping syndrome and weight loss (3). Pylorus-preserving gastrectomy (PPG) or proximal gastrectomy (PG) has been demonstrated with promising benefits with less dumping syndrome and weight loss. However, for PGC, the choice of TG or PG has been a controversial issue (4, 5). In fact, the fundamental difference between PG and TG is the extent of lymph node dissection. Whether to clean the No. 5 and No. 6 lymph nodes in the operation of PGC fundamentally determines the choice of surgical approach. The Japanese Gastric Cancer Association (JGCA) divided the lymph nodes into 4 levels according to the primary location of the gastric tumor based on the lymphatic flow and other important parameters (6). Guidelines for the diagnosis and treatment of gastric cancer in Japan suggested that all gastric cancer patients with T_1_N+ and T2–T4 stage should accept standard TG and D2 lymph node dissection for the purpose of radical treatment (6). However, compared to PG patients, those undergoing TG perform worse on postoperative nutritional indicators (5). This raises a question: whether all PGC patients require standard TG+D2 lymph node dissection. In other words, whether all patients with PGC need to receive No. 5 and No. 6 lymph node dissection or not. The likelihood of No. 5 and No. 6 lymph node plays a pivotal role in considering the extension of lymphadenectomy, which will further determine the preservation of pylorus.

In view of the above, this study will analyze the metastases of lymph node groups in patients with PGC and further analyze the metastases of No. 5 and No. 6 lymph nodes and risk factors, so as to investigate the necessity of the No. 5 and No. 6 lymph node dissection in PGC patients.

## Methods

### Study Population

The clinical data of 1,734 patients, who had undergone radical gastrectomy in the Department of Gastrointestinal Surgery, Ruijin Hospital, Shanghai Jiao Tong University School of Medicine from January 2008 to December 2017 were retrospectively reviewed. The exclusion criteria were as follows: 1) who received neoadjuvant therapy; 2) tumor center, located in the middle or lower third of the stomach or pathological report could not clearly indicate the lymph node dissection; 3) pathological findings of distant metastases; 4) pathological type of squamous cell carcinoma.

### Treatment Measures

All patients enrolled in the study underwent radical TG + D2/D2 + lymph node dissection and met the criteria for R0 resection according to standards established by JGCA (6). Digestive reconstruction was performed using the Roux-en-Y procedure.

### Data Collection

The tumor location was determined according to the location of the central point of the tumor recorded in the pathological report. The PGC patients were divided into two groups: esophago-gastric junction (EGJ) group and stomach body group. All EGJ tumors were located within 2 cm of the esophago-gastric junction. The lymph node tissues dissected during operation were strictly operated in accordance with the paraffin pathological biopsy. The number of lymph node dissection and the number of metastases were recorded in the postoperative pathology report. The pathology reports with unclear group or missing lymph node count were recorded as “incomplete” and removed from the research. Due to the limitations of our technique to separate lymph nodes in operating room and pathology department, the No. 7, No. 8, and No. 9 lymph nodes were counted as a group of lymph nodes in this study in agreement. Tumor depth was pathologically classified into four groups as T1, T2, T3, and T4 according to the eighth edition of the AJCC TNM staging system (7). The pathological type of tumor was classified according to the presence or absence of signet ring cell carcinoma. Poorly differentiated adenocarcinomas and signet ring cell carcinomas were classified as poorly differentiated tumors. Well and moderately differentiated tubular adenocarcinoma and papillary adenocarcinoma were grouped together as well/moderate differentiated tumors. Patient data were classified and compared by age, gender, Borrmann type, tumor size, and perigastric LNM except No. 5 and No. 6 lymph nodes.

### Statistical Analysis

All data were analyzed by SPSS (IBM SPSS software version 25.0) statistical analysis. The χ^2^ test was used for clinicopathological characteristic comparison and univariate analysis. Logistic regression analysis was used for multivariate analysis. P < 0.05 was considered statistically significant.

## Results

### Study Population

**Figure 1:**
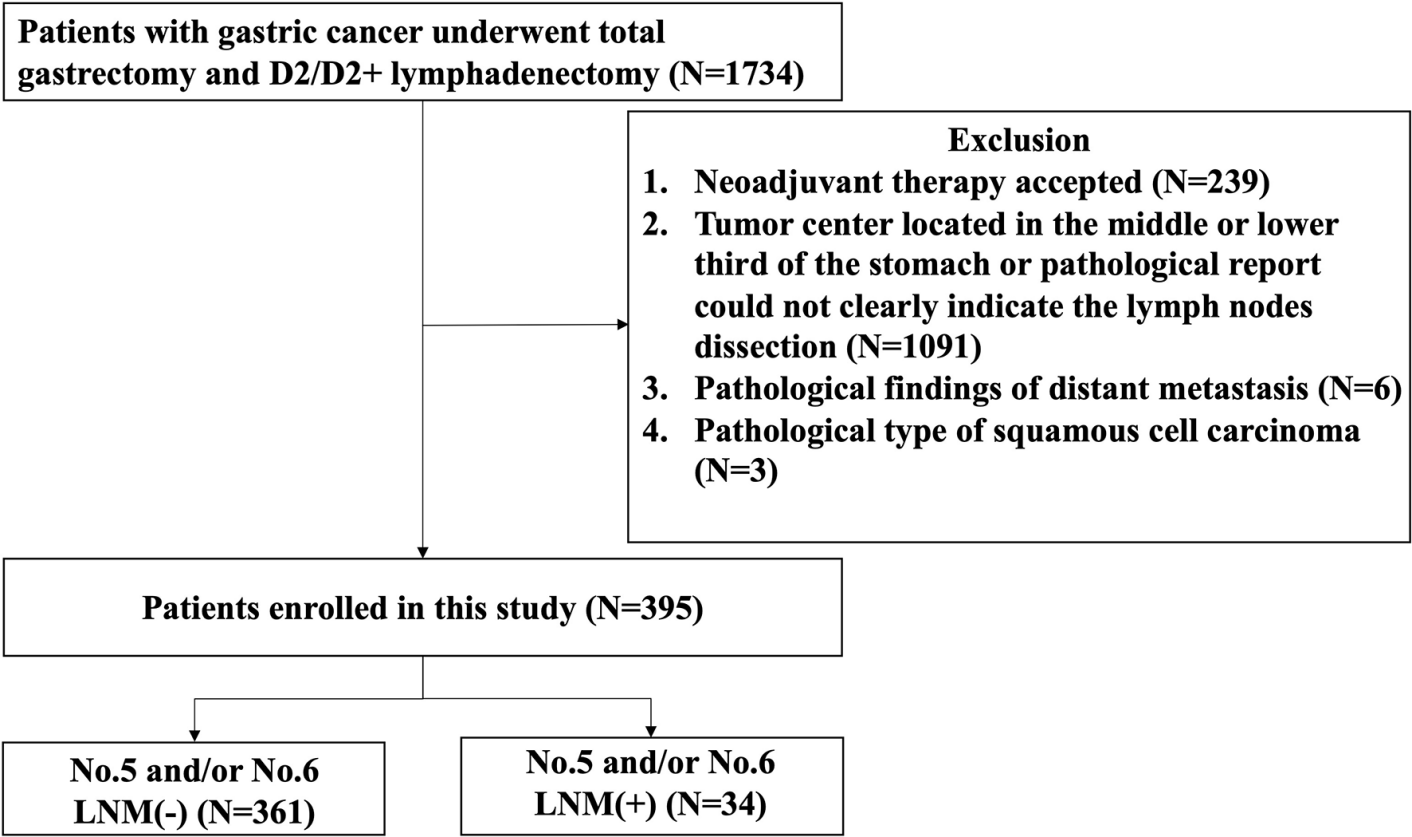
Flowchart of patient selection.

The flow diagram of the patients eligible for this study is shown in **Figure 1**. The number of patients, who underwent radical gastrectomy with TG and D2/D2 + lymphadenectomy, included in this study was 1,734, of whom 239 patients were excluded for receiving neoadjuvant therapy, 1,091 for tumor center located in the middle or lower third of the stomach or pathological report not clearly indicate the lymph node dissection, 6 for distant metastasis, and 3 for squamous cell carcinoma. Finally, a total of 395 eligible patients were enrolled in this study, of whom 361 patients without No. 5 and/or No. 6 LNM and 34 patients with No. 5 and/or No. 6 LNM.

### Incidence of No. 5 and/or No. 6 Lymph Node Metastasis in Proximal Gastric Cancer Cases

**Figure 2:**
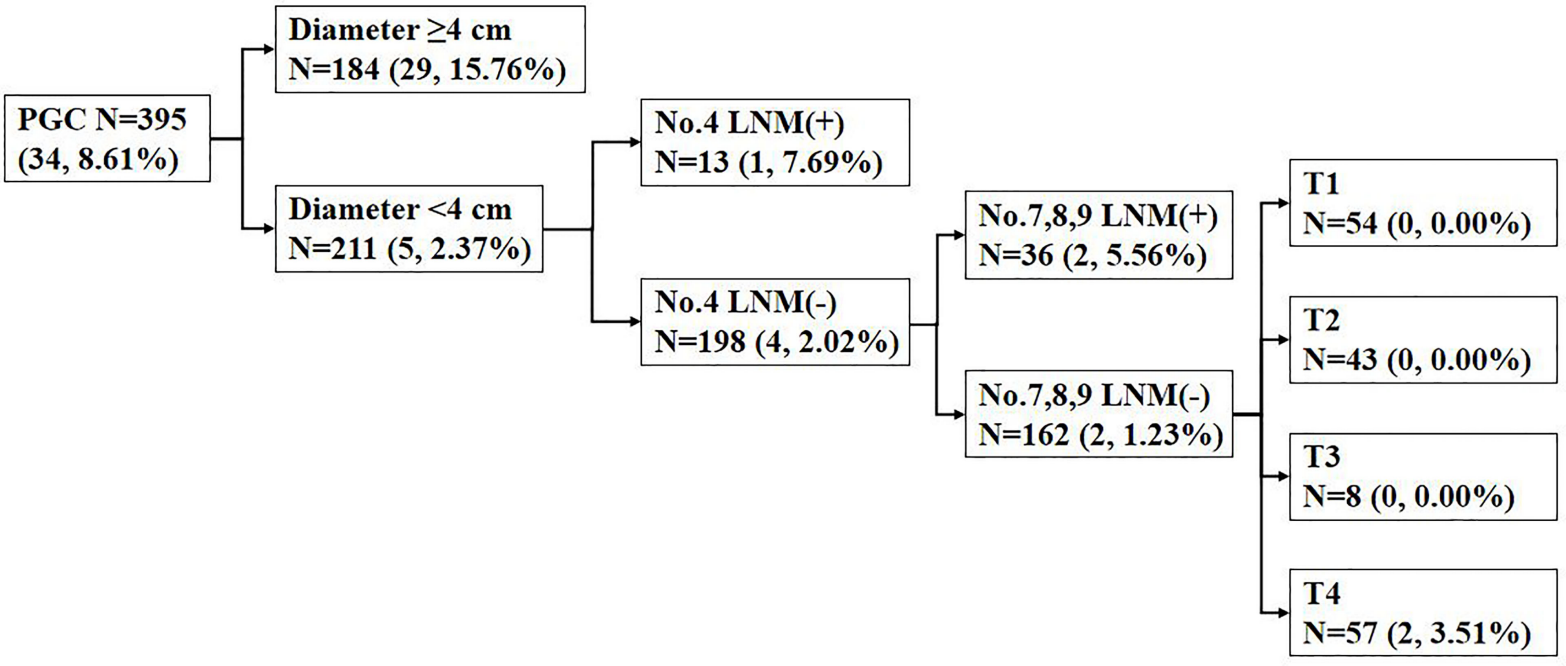
The incidence of No. 5 and/or No. 6 lymph node metastasis (LNM) in proximal gastric cancer (PGC) cases according to the diameter; No. 4 LNM; No. 7, No. 8, No. 9 LNM; and T stage. Data are presented as N (n, %). LNM, lymph node metastasis.

Among the 395 patients with PGC, 184 patients developed tumor diameter ≥4 cm with No. 5 and/or No. 6 LNM rate of 15.76%, while 211 patients were with tumor diameter <4 cm and No. 5 and/or No. 6 LNM rate of 5/211 (2.37%). Among patients with diameter <4 cm, No. 4 LNM positive rate was 7.69%, while in patients without No. 4 LNM, 4/198 (2.02%) patients showed positive No. 5 and/or No. 6 LNM. Among the No. 4 LNM (-) patients, No. 5 and/or No. 6 LNM (+) was observed in 2/36 (5.56%) No. 7, No. 8, No. 9 LNM (+) patients and in 2/162 (1.32%) No. 7, No. 8, No. 9 LNM (-) patients. Among the No. 7, No. 8, No. 9 LNM (-) patients, the patient distribution was 54 in T1 stage, 43 in T2 stage, 8 in T3 stage, and 57 in T4 stage (**Figure 2**).

### Overall Metastases of Perigastric Lymph Nodes

Among the 395 patients with PGC, lymph node metastases were found in 242 patients (61.27%), with an average of 33.68 ± 13.20 (range from 10 to 98) lymph nodes retrieved and 4.67 LNMs per patient. **Table 1** showed the overall LNM of 395 patients grouped according to the T stage. The incidence rate of LNM increased with the upgrade of T stage, and the incidence rates among the four groups are significantly different (T1: 16.67%, T2: 39.39%, T3: 60.00%, T4: 79.83%, χ^2^ = 103.212, P < 0.001). In addition, the incidence rate of LNM from high to low were No. 3 (45.06%); No. 1 (31.65%); No. 7, No. 8, No. 9 (27.85%); No. 2 (21.01%); No. 4 (13.42%); No. 6 (6.08%); and No. 5 (4.81%). The metastatic rate of No. 5 and/or No. 6 was 8.61%, significantly lower than that of other perigastric lymph nodes (**Table 1**).

**Table 1:** Overall lymph node metastatic rate according to tumor depth of invasion.

Lymph Group	Lymph node metastatic rate				Total	χ^2^ value	P-value
	Tumor depth of invasion						
	T1	T2	T3	T4			
No. 1	4/66 (6.06)	8/66 (12.12)	5/25 (20.00)	108/238 (45.38)	125/395 (31.65)		
No. 2	2/66 (3.03)	8/66 (12.12)	3/25 (12.00)	70/238 (29.41)	83/395 (21.01)		
No. 3	6/66 (9.09)	16/66 (24.24)	12/25 (48.00)	144/238 (60.50)	178/395 (45.06)		
No. 4	3/66 (4.55)	2/66 (3.03)	2/25 (8.00)	46/238 (19.33)	53/395 (13.42)		
No. 5	0/66 (0.00)	1/66 (1.52)	0/25 (0.00)	18/238 (7.56)	19/395 (4.81)		
No. 6	1/66 (1.52)	0/66 (0.00)	1/25 (4.00)	22/238 (9.24)	24/395 (6.08)		
No. 7, No. 8, No. 9	6/66 (9.09)	8/66 (12.12)	6/25 (24.00)	90/238 (37.82)	110/395 (27.85)		
No. 5 and/or No. 6	1/66 (1.52)	1/66 (1.52)	1/25 (4.00)	31/238 (13.03)	34/395 (8.61)		
Total	11/66 (16.67)	26/66 (39.39)	15/25 (60.00)	190/238 (79.83)	242/395 (61.27)	103.212	**<0.001**

Data are presented as n/N (%).

The χ^2^ test was used for analysis of perigastric LNM among different tumor depths of invasion. Bold value means there were significantly differences in those variables because the P value is less than 0.05.

### Univariate Analysis of No.5 and/or No.6 Lymph Node Metastasis

The basic clinical features of the eligible patients were listed in **Table 2**. The No. 5 and/or No. 6 LNM was found in 8.61% patients (n = 34). There was no significant difference in gender and age. In addition, univariate analysis identified that depth of invasion (χ^2^ = 15.294, P = 0.001), tumor diameter (≥4 cm) (χ^2^ = 25.405, P < 0.001), degree of differentiation (χ^2^ = 4.600, P = 0.034), Borrmann type (χ^2^ = 17.492, P = 0.004), vascular or perineural invasion (χ^2^ = 7.845, P = 0.006), and the other perigastric LNMs except No. 5 and No. 6 (χ^2 =^ 17.363, P < 0.001) were risk factors for No. 5 and/or No. 6 LNM. 

**Table 2:** Clinicopathological features and univariate analysis of potential risk factors of No. 5 and/or No. 6 LNM.

Variables	Positive rates of No. 5 and/or No. 6 LNM^#^	χ^2^ value	P-value
Gender		0.440	0.547
Male	27/295 (9.15)		
Female	7/100 (7.00)		
Age (year)		<0.001	0.995
<60	10/116 (8.62)		
≥60	24/279 (8.60)		
Depth of invasion (T stage)		15.294	**0.001**
T1	1/66 (1.52)		
T2	1/66 (1.52)		
T3	1/25 (4.00)		
T4	31/238 (13.03)		
Lesion location		1.264	0.282
esophago-gastric junction	16/222 (7.21)		
Stomach body	18/173 (10.40)		
Tumor diameter (cm)		25.405	**<0.001**
<4	5/211 (2.37)		
≥4	29/184 (15.76)		
Pathological types		3.548	0.072
Adenocarcinoma	23/316 (7.28)		
Signet ring cell carcinoma	11/79 (13.92)		
Degree of differentiation		4.600	**0.034**
Well/Moderate differentiation	3/94 (3.19)		
Poor differentiation	31/301 (10.30)		
Borrmann type		17.492	**0.004**
Early gastric cancer (T1)	1/66 (1.52)		
I	2/43 (4.65)		
II	0/13 (0.00)		
III	25/252 (9.92)		
IV	6/21 (28.57)		
Vascular or perineural invasion		7.845	**0.006**
Negative	11/218 (5.05)		
Positive	23/177 (12.99)		
Other perigastric LNM		17.363	**<0.001**
Negative	2/155 (1.29)		
Positive	32/240 (13.33)		

^#^Data are presented as n/N (%). LNM, lymph node metastasis.

The χ^2^ test was used for univariate analysis of potential risk factors and rate of No. 5 and/or No. 6 LNMs. Bold value means there were significantly differences in those variables because the P value is less than 0.05.

Further analysis indicated that the incidence rate of No. 5 and/or No. 6 LNM among the T1 (1.52%), T2 (1.52%), and T3 (4.00%) groups showed no significant difference, while, when compared with that of the T4 group (13.03%), the difference showed statistical significance (P = 0.001). As for other perigastric lymph nodes, except No. 5 and No. 6 LNM, univariate analysis indicated that all of other perigastric LNMs were significantly associated with a higher rate of No. 5 or No. 6 LNM (**Tables 2**, **3**).

**Table 3:** The analyses of relationship between No. 5 and/or No. 6 LNM and other perigastric LNMs.

	Positive rates of No. 5 and/or No. 6 LNM^#^	χ^2^ value	P-value
No. 1		45.504	**<0.001**
Negative	0/270 (0.00)		
Positive	20/125 (16.00)		
No. 2		15.206	**<0.001**
Negative	18/312 (5.77)		
Positive	16/83 (19.28)		
No. 3		24.322	**<0.001**
Negative	5/217 (2.30)		
Positive	29/178 (16.29)		
No. 4		50.023	**<0.001**
Negative	16/342 (4.68)		
Positive	18/53 (33.96)		
No. 7, No. 8, No. 9		38.637	**<0.001**
Negative	9/285 (3.16)		
Positive	25/110 (22.73)		

^#^Data are presented as n/N (%). LNM, lymph node metastasis.

The χ^2^ test was used for relationship analyses between the rate of No. 5 and/or No. 6 LNM and other perigastric LNMs. Bold value means there were significantly differences in those variables because the P value is less than 0.05.

### Multivariate Analysis of No. 5 and/or No. 6 Lymph Node Metastasis

Among the risk factors selected from the univariate analysis, tumor diameter ≥4 cm (OR = 4.104, 95% CI 1.331–12.653, P = 0.014), No. 4 LNM (OR = 4.642, 95% CI 1.750–12.312, P = 0.002), and No. 7, No. 8, No. 9 LNM (OR = 4.606, 95% CI 1.773–11.967, P = 0.002) remained significant in multivariate analysis (**Table 4**). These were independent risk factors for No. 5 and/or No. 6 LNM.

**Table 4:** Multivariate logistic regression analyses for No. 5 and/or No. 6 LNM.

Variables	OR (95% CI)	P-value
Constant	0.002	<0.001
Gender	0.517 (0.168-1.590)	0.250
Age (≥60 years old)	1.690 (0.600-4.762)	0.321
Depth of invasion (T stage)	1.300 (0.612-2.762)	0.496
Lesion location	1.737 (0.722-4.176)	0.218
Tumor diameter (≥4 cm)	**4.104 (1.331-12.653)**	^**^0.014
Pathological types	1.415 (0.500-4.003)	0.513
Degree of differentiation	1.328 (0.317-2.266)	0.698
Borrmann type	1.199 (0.660-2.179)	0.551
Vascular or perineural invasion	1.133 (0.448-2.865)	0.792
No. 1 LNM Positive	1.277 (0.487-3.349)	0.620
No. 2 LNM Positive	0.650 (0.237-1.782)	0.402
No. 3 LNM Positive	1.692 (0.517-5.539)	0.385
No. 4 LNM Positive	**4.642 (1.750-12.312)**	^**^0.002
No. 7, No. 8, No. 9 LNM Positive	**4.606 (1.773-11.967)**	^**^0.002

^**^P < 0.05 was significant.

95% CI, 95% confidence interval; LNM, lymph node metastasis.

Logistic regression analysis was used for multivariate analysis for No. 5 and/or No. 6 LNM. Bold value means there were significantly differences in those variables because the P value is less than 0.05.

### Lymph Node Metastasis Rate of Adenocarcinoma With Tumor Diameter <4 cm According to Location in Esophago-Gastric Junction or Stomach Body

LNM rate of adenocarcinoma in patients with tumor diameter <4 cm was also analyzed. According to the site of tumor location in EGJ or stomach body, metastatic rate of lymph node No. 5, No. 6, and No.5/No.6 were 0.92%, 1.83%, and 2.75%, and 0.98%, 1.96%, and 1.96% in EGJ and stomach body, respectively (**Table 5**). The LNM rates of lymph node No. 1, No. 2, No. 3, No. 4, and No. 7, No. 8, No. 9 in EGJ and stomach body were also shown in **Table 5**.

**Table 5:** LNM rate of adenocarcinoma with tumor diameter <4 cm according to location in EGJ or stomach body.

Variables	LNM rate of adenocarcinoma (<4 cm)	
	EGJ	Stomach body
No. 1	28/109 (25.69%)	19/102 (18.63%)
No. 2	9/109 (8.26%)	11/102 (10.78%)
No. 3	30/109 (27.52%)	35/102 (34.31%)
No. 4	2/109 (1.83%)	11/102 (10.78%)
No. 5	1/109 (0.92%)	1/102 (0.98%)
No. 6	2/109 (1.83%)	2/102 (1.96%)
No. 5 and/or No.6	3/109 (2.75%)	2/102 (1.96%)
No. 7, No. 8, No. 9	24/109 (22.02%)	20/102 (19.61%)

Data are presented as n/N (％).

EGJ, esophago-gastric junction; LNM, lymph nodes metastasis.

## Discussion

In this study, we retrospectively enrolled eligible PGC patients who underwent TG and D2/D2 + lymphadenectomy and found that the positive rate of No. 5 and No. 6 LNM was low but increasing with the upgrade of T stage. Besides tumor size, other perigastric LNMs, i.e., No. 4 LNM and No. 7, No. 8, No. 9 LNM, are independent risk factors for No. 5 and/or No. 6 LNM. The findings of this study provide evidence for the feasibility of preserving No. 5 and No. 6 lymph node in PG for some T1–T3 PGC patients because of low risk of No. 5 and/or No. 6 LNM.

LNM of gastric cancer is not only a manifestation of tumor nature, but also one of the main factors associated with the prognosis of gastric cancer (8–11). Besides, the rule of LNM is the basis of the lymph node dissection in radical surgery for gastric cancer. The scope of a radical resection of gastric cancer can be roughly divided into two parts: the gastrectomy and the lymphadenectomy. These two parts are closely related. For example, the No. 5 and No. 6 lymph nodes cannot be resected easily without resecting the right gastroepiploic vessels below the pylorus and the right gastric vessels above the pylorus, which means that it is too difficult to resect the No. 5 and No. 6 lymph nodes during a PPG or PG. This might be one of the reasons why patients undergoing PG have a higher long-term recurrence rate than those undergoing TG (12, 13). On the other hand, PG is better than TG on long-term maintenance of nutrition and quality of life (5, 14, 15). Researchers report that patients undergoing PG have similar (14, 16) or even longer (17) survival time after surgery compared to TG. Hence, it is necessary to identify those patients with low risk of No. 5 and No. 6 LNM who can safely undergo PG instead of TG.

In this retrospective study, the metastasis rate of the No. 5 and No. 6 lymph node was the lowest (1.52%–13.03%) in every T stage subgroup and lower than the No. 7, No. 8, and No. 9 (9.09%–37.82%), which used to be regarded as the second station lymph node in PGC. The finding was consistent with previous studies that indicated that the rate of No. 5 and No. 6 LNM was lower in the PGC patients compared with other perigastric lymph nodes (8, 18–24). However, there are few reports on the analysis of specific risk factors for No. 5 and No. 6 LNM rate in PGC patients.

We noticed that the location of the lesion is not a risk factor of No. 5 or No. 6 LNM in PGC. In recent years, researchers tried to find out the relationship between lesion location and LNM rate. Han et al. (25) demonstrated that the metastasis rate was 0.6% for No. 5 and 1.9% for No. 6 in PGC patients. However, they did not analyze the difference between EGJ region and the rest of the upper one-third of the stomach. Thereafter, the No. 5 and No. 6 lymph nodes also showed a low metastasis rate (<1%) regardless of T stage if the diameter was <4 cm in EGJ adenocarcinoma (26). Thus, it can be seen that the prophylactic dissection of the No. 5 and No. 6 lymph nodes is of limited significance and questionable in patients with tumors located in the upper one-third of the stomach (26–30).

As we can learn from the JGCA guidelines (6), the dissection of No. 5 and No. 6 is not required when treating EGJ tumor smaller than 4 cm because of the low risk of LNM but still needed for T1N+ and advanced gastric cancer. In our study, for patients with tumor diameter <4 cm, the rates of No. 5 and/or No. 6 LNM were 1.96% and 2.75% with tumors located in stomach body and EGJ region. Since the patterns of No. 5 and No. 6 LNM were so similar, whether tumor located in EGJ and the rest of the proximal stomach should be treated differently when performing lymphadenectomy needs further study.

For early PGC, it has been documented that, when compared with TG, the radical PG has no significant difference on long-term outcomes (5, 15, 31–33), which suggests that the preservation of No. 5 and No. 6 lymph nodes in the surgical treatment of early gastric cancer is not a determinant of prognosis due to the low risk of No. 5 and No. 6 LNM in early PGC. The metastasis rates of No. 5 and/or No. 6 lymph nodes in T1 was 1.52% in our research. In our research, the T stage was a risk factor for No. 5 and/or No. 6 LNM, but there was no significant difference in the rate of No. 5 and/or No. 6 LNM among T1, T2, and T3 patients with proximal gastric adenocarcinoma. This indicated that it is possible for some T2 and T3 patients to receive a No. 5 or No. 6 lymph node-preserving as well as T1 PGC patients. Yun et al. (21) showed that T stage can be an independent risk factor of No. 5 and No. 6 LNM in their research. However, T stage is not an independent risk factor in our study, which may be attributed to the low rate of No. 5 and/or No. 6 LNM and the lack of positive objects in T1–T3 patients. Though a low rate of No. 5 and/or No. 6 LNM in patients with PGC <4 cm and other perigastric LNM(-) (especially No. 4 and No. 7, No. 8, No. 9) is observed in our study (**Figure 2**), it is still risky to preserve No. 5 and No. 6 lymph nodes for T4 patients. In short, in our study, we think that it is not advisable to preserve No. 5 and No. 6 lymph nodes in T4 patients because the rate of No. 5 and/or No. 6 LNM was significantly higher in univariate analysis (**Table 2**) and subsequent analysis (**Figure 2**) compared with T1–T3 patients.

Tumor diameter ≥4 cm was one of the independent risk factors of No. 5 and/or No. 6 LNM in our study. Researchers previously indicated that tumor size might be a relevant risk factor for LNM regardless of early or advanced gastric cancer. Tumor size <8 cm was earlier found to be a risk factor of LNM (34), and tumor size <2 cm was identified as a criterion for risk factors afterward (25). This bias might be due to the differences in the target population: the study population of the latter (35) is early PGC patients, while the earlier study did not specify a specific site or T stage (34). Other researchers, e.g., Khalayleh et al. (19) and Yun et al. (21) reported that tumor size <4.1 cm or <5 cm also leads to a lower rate of No. 5 and/or No. 6 LNM in their own research. But in general, it is believed that larger tumor size is associated with higher risk of LNM. In our study, the standard was 4 cm, which was close to JGCA guidelines (6). Tumor size can be measured easily before or during surgery, which made it a valuable predictive factor of No. 5 and/or No. 6 LNM.

In this study, other perigastric LNMs, especially No. 4 and No. 7, No. 8, No. 9 LNMs, were independent risk factors for No. 5 and/or No. 6 LNM. The system of lymphatic drainage proposed by Rouvière (36) has been confirmed as a whole. So, it is well known that the No. 6 lymph nodes directly received drainage from No. 4 lymph nodes, while No. 7, No. 8, No. 9 lymph nodes can be drained from No. 5 and No. 6 lymph nodes. Generally speaking, the No. 4 and No. 7, No. 8, No. 9 LNMs are valuable to access the possibility of No. 5 and No. 6 LNMs. But as shown in **Table 3**, every group of perigastric LNM was significantly associated with a higher rate of No. 5 and/or No. 6 LNM. Thus, we recommend that patients with perigastric LNM (not only No. 4 or No. 7, No. 8, No. 9) should undergo dissection of No. 5 and No. 6 lymph nodes. Intraoperative frozen section analysis is a powerful tool to identify metastatic lymph nodes during the surgery. The finding in this retrospective study was in line with the result of another study, in which patients with lymph node No. 4 positive, tumor size ≥5 cm, and T4 stage are recommended for lymphadenectomy with No. 5 and No. 6 and TG (37).

Does the extremely low rate of No. 5 and No. 6 LNM suggest that resection of these lymph nodes is unnecessary? Sasako et al. (38) reported an index known as the index of estimated benefit from lymph node dissection (IEBLD) to evaluate the therapeutic value of lymph node dissection for gastric cancer in 1995. The index is calculated by multiplying the metastatic rate of a certain lymph node station by the 5-year survival rate of corresponding metastatic patients. IEBLD = 0 is defined as no value in lymph node dissection. IEBLD >0 is defined as having a therapeutic value for lymph node dissection. And the higher the index, the more beneficial for lymphadenectomy. Ri et al. (20) reported that IEBLD of No. 5 and No. 6 lymph nodes was zero or extremely low in cT2–T4 lesions located within the cardia and/or the fornix. Fujitani et al. (23) and Cao et al. (24) recommended that Siewert II AEG patients might benefit from PG plus limited lymphadenectomy because of the low IEBLD of No. 5 and No. 6 lymph nodes. Due to the lack of survival data, we failed to provide our own IEBLD data in this research. However, with our data showing extremely low rates of No. 5 and No. 6 LNMs, we estimate that IEBLD of No. 5 and No. 6 lymph nodes will not be high; dissection of No. 5 and No. 6 lymph nodes is not essential for T1–T3 PGC patients without perigastric LNM (especially No. 4 and No. 7, No. 8, No. 9), whose rate of No. 5 and/or No. 6 LNM is zero in our study.

There are some limitations in our study: Firstly, this study is a single-center retrospective study with unavoidable bias (e.g., selection bias) and data shortage. Secondly, restricted by the technique of separating lymph nodes in operating room and pathology department, no further analysis was performed in this study for No. 7, No. 8, No. 9 lymph nodes and other stations, such as No. 10 and No. 11. Thirdly, this study did not involve the analysis of the prognosis and survival data. High-quality randomized controlled trials are still expected to elucidate the real worth of PG and TG.

## Conclusion

The rate of No. 5 and/or No. 6 LNM is extremely low in T1–T3 stage PGC patients with tumors <4 cm and without other perigastric LNMs (especially No. 4 and No. 7, No. 8, No. 9). It is feasible to preserve No. 5 and No. 6 lymph nodes with PG for these patients rather than TG.

## Data Availability Statement

The raw data supporting the conclusions of this article will be made available by the authors without undue reservation.

## Ethics Statement

The studies involving human participants were reviewed and approved by Ruijin Hospital Ethical Committees, Shanghai Jiao Tong University School of Medicine. Written informed consent for participation was not required for this study in accordance with the national legislation and the institutional requirements.

## Author Contributions

CL and YZ contributed to conception and design of the study. XY and YZ organized the database and performed the statistical analysis. XY and YZ wrote the first draft of the article. RF, ZZ and MY provided many advices about the writing of the article. All authors contributed to article revision and read and approved the submitted version.

## Conflict of Interest

The authors declare that the research was conducted in the absence of any commercial or financial relationships that could be construed as a potential conflict of interest.

## Publisher’s Note

All claims expressed in this article are solely those of the authors and do not necessarily represent those of their affiliated organizations, or those of the publisher, the editors and the reviewers. Any product that may be evaluated in this article, or claim that may be made by its manufacturer, is not guaranteed or endorsed by the publisher.
